# Types and Presentation of Refractive Error among Individuals Aged 0–30 Years: Hospital-Based Cross-Sectional Study, Yemen

**DOI:** 10.1155/2021/5557761

**Published:** 2021-07-05

**Authors:** Tawfik Saleh Mohammed Dhaiban, Femina Purakaloth Ummer, Hanan Khudadad, Shajitha Thekke Veettil

**Affiliations:** ^1^Department of Operations, Al Thumama Health Centre, Primary Health Care Corporation, Doha, Qatar; ^2^Department of Operations, Airport Health Centre, Primary Health Care Corporation, Doha, Qatar; ^3^Clinical Research Department, Primary Health Care Corporation, Doha, Qatar

## Abstract

**Background:**

Refractive errors are the most common cause of visual impairment worldwide. Its proportion varied among societies and is considered as a public health challenge. Symptoms and signs associated with refractive errors are the most worrisome and common presentations in the general practice in eye clinics.

**Aim:**

The goal of this study was to determine the types and presentations of refractive error among the 0–30-year-old Yemeni population to aid early identification, diagnosis, referral, and treatment. *Methodology*. A cross-sectional study including 1,500 out-patients aged from 0 to 30 years attending the ophthalmology clinic in Sanaa, Yemen (between 2012 and 2015). All patients underwent visual acuity examination, autorefractometer, and anterior and posterior segment examination and were grouped according to type, that is, myopia, hyperopia, and astigmatism.

**Results:**

Hyperopia was the most common single diagnosis (53.3%) followed by myopia (33.3%). Astigmatism was uncommon as a single diagnosis (13.4%) but commonly associated with hyperopia or myopia. Myopia was more common in males (42.9%) than in females (25%). Hyperopia was more in females (62.5%) than in males (42.9%). Age groups most affected by refractive errors were 13–18 years (27.7%), 19–24 years (24.8%), and 25–30 years (24.6%), respectively. Decreased vision (53%) was a common presentation in myopia and astigmatism (41.5%) and less in hyperopia (39.6%). Headache was common in astigmatism (56%), hyperopia (28.8%), and myopia (17.8%). Muscle imbalance, namely, exotropia (27.2%), is mainly found in myopia and esotropia (24.3%) in hyperopia.

**Conclusions:**

In addition to decreased vision, our patients with refractive errors mostly complain of headaches with clear variations with age and type of refractive error. Early identification and proper categorization of refractive errors by age, gender, and other demographics by general physicians in primary care can better deduce and make useful referrals to eye specialists.

## 1. Introduction

Refractive error is a problem with focusing light accurately on the retina due to the shape of the eye. The most common types of refractive error are near-sightedness (myopia), far-sightedness (hyperopia), astigmatism, and presbyopia. Near-sightedness results in faraway objects being blurry, far-sightedness, presbyopia results in close objects being blurry, and astigmatism causes objects to appear stretched out or blurry. Other symptoms may include double vision, headaches, and eye strain. Refractive errors are corrected with eyeglasses, contact lenses, or surgery [1]. The number of people globally with refractive errors has been estimated at one to two billion [2]. Rates vary between regions of the world with about 25% of Europeans and 80% of Asians affected [2]. Myopia is the most common disorder [3]. Rates among adults are between 15 and 49% while rates among children are between 1.2 and 42% [4]. Hyperopia more commonly affects young children and the elderly [5, 6]. Presbyopia affects most people over the age of 35 [1]. In 2013, 660 million people (10 per 100) were estimated with refractive errors that have not been corrected. Of these, 9.5 million were blind due to the refractive error [7]. Refractive error is one of the most common causes of blindness along with cataracts, macular degeneration, and vitamin A deficiency [8].

Decreased vision and headache are common presentations associated with visual disability and refractive errors seen in general practice and at specialist eye clinics [9–11]. Myopia is a global public health problem affecting all age groups. A few epidemiological studies have shown variations within and between countries [12]. For instance, in studies in Asian countries, the prevalence in adults varies between 19.4% in Taiwan [13] to 48.1% in Indonesia [14]. In North America and Europe, prevalence varies from 33.1% in the USA [15] to as high as 49% among 40-year-old adults in the United Kingdom [15]. Worldwide variations of myopia in children also show similar differences across countries and regions as shown from the results of the Refractive Error Study in Children [16] which was conducted in different countries using the same sampling strategies and procedures to measure refraction, and similar definitions of myopia to enable intercountry comparisons [16]. The study found differences between urban and rural children in Nepal with a progressive increase in prevalence from 5 to 15 years. In India, the prevalence appeared to decrease from rural to urban children whereas, among urban children in China, the prevalence of myopia varied from 5.7% in 5-year-old to 30.1% in 10-year-old to 78.4% in 15-year-old people [17, 18].

According to the WHO regions, the estimated pool prevalence (EPP) of hyperopia was 4.6% (95% CI: 3.9*e*5.2) in children. The lowest and highest EPP was seen in South-East Asia (2.2%, 95% CI: 1.2*e*3.3) and the Americas (14.3%, 95% CI: 13.4*e*15.2), respectively. The EPP of hyperopia was 30.6% (95% CI: 26.1*e*35.2) in adults. Based on the results of the meta-analysis, Africa had the highest EPP of hyperopia (38.6%, 95% CI: 22.4*e*54.8) followed by the Americas (37.2%, 95% CI: 25.3*e*49) while Europe had the lowest EPP (23.1%, 95% CI: 6.1*e*40.2) [19].

The definition of astigmatism in epidemiologic studies has less variation. Considering the changes of astigmatism with age, in children above 1 year old, astigmatism was 14.9% (95% CI: 12.7*e*17.1). According to WHO regions, the lowest estimated pool prevalence (EPP) was seen in South-East Asia (9.8%) while the highest EPP was seen in the Americas (27.2%) followed by the Eastern Mediterranean region (20.4%). For adults, studies showed that 40.4% (95% CI: 34.3*e*46.6) of adults had astigmatism. However, astigmatism showed a lot of variation in different WHO regions; the highest EPP astigmatism was seen in the Americas, and the lowest EPP was seen in Africa (11.4% vs. 45.6%). However, it should be noted that only one study was conducted in the Americas. After the Americas, South-East Asia had the highest EPP of astigmatism (44.8%, 95% CI: 36.6*e*53.1) [19].

Given how common these eye defects are in clinical practice, research that helps to understand their natural history and especially local risk factors will be helpful for early diagnosis and properly targeted treatments in primary care practice. Therefore, the focus of this study was to determine the types and presentations of refractive errors among the 0–30-year-old Yemeni population a basis for developing preventive interventions.

## 2. Methods

### 2.1. Study Design and Setting

This was a cross-sectional clinical epidemiological study involving a convenient purposive sample of patients referred from across the country for ophthalmic consultation at the department of ophthalmology, Saudi–German Hospital, a major referral center for eye problems in Sanaa, Yemen. During the study period, all patients with eye problems are referred to the Saudi–German Hospital due to the limited facilities across the health centers in Yemen.

### 2.2. Participants

This was a purposive sample whose selection was based on their willingness to participate in the study once they met the inclusion criteria. Given that our primary goal was to determine the types and presentation of refractive errors in our practice population, no specific sample size was calculated. No other similar studies were reported from the area on this topic.

### 2.3. Sample Size and Categorization

A total of 1500 patients between ages 0 and 30 years were included in this study following informed consent. Subjects were divided into the following age groups 0–6, 7–12, 13–18, 19–25, and 26–30 in order to include the maximum patients who agreed to participate in the study. We identified the patients who can benefit from vision rehabilitation. Then, we accurately measure visual acuity and provide appropriate vision rehabilitation services. Eligibility included referral for ophthalmic consultation by other clinicians for symptoms including decreased vision, headaches, esotropia, and exotropia. Patients, with a history of eye injury, aphakia retinoblastoma, and systemic or congenital disease were excluded from the study.

### 2.4. Data Sources/Measurement

Procedures: demographic data were obtained from patients' records and/or referral notes. All patients underwent visual acuity assessment and refraction measurement by cycloplegic autorefraction and subjective refraction.

Visual Acuity Testing was performed with tumbling *E*, at a distance of 6 m. The right eye was tested first and then the left, each time with occlusion of the fellow eye. Ocular Examinations were done by a trained team consisting of an ophthalmologist. After assessment of visual acuity as above, axial length was measured by an IOL Master (version 5.02; Carl Zeiss, Jena, Germany), and a slit lamp (YZ5X; 66 Vision Tech, Suzhou, China) examination and direct ophthalmoscopy were performed by an ophthalmologist. Intraocular pressure was measured by an optometrist using noncontact tonometry (NT-1000; Nidek, Tokyo, Japan), and participants having a peripheral anterior chamber depth of >1/2 the thickness of cornea, IOP 25 mm Hg, given cycloplegia of one drop of 0.5% proparacaine hydrochloride in each eye followed by two drops of cyclopentolate 1.0% (Cyclogyl; Alcon, Fort Worth, TX, USA) 5 minutes apart. If the pupil size was ‡6 mm and the light reflex was absent after 30 minutes, cycloplegia was deemed adequate. Subsequently, autorefraction (KR-8900; Topcon, Tokyo, Japan) and subjective refraction were performed by an experienced optometrist.

A value of 6/6 or 20/20 is considered optimum, or perfect vision. Individuals who have 20/20 vision are able to read letters that are 3/8 of an inch tall from 20 feet away. Those who do not have 20/20 vision are considered as refractive error. People who require spherical equivalent (SE) ≤ −0.50 Diopters of optical correction are considered as having myopia, SE ≥ +2.0. Diopters are considered having hyperopia and SE of −2.00 + 3.50 × 095. Diopters are considered having astigmatism. Once examined, patients were grouped into the following five categories: myopia only, hyperopia only, astigmatism only, myopia with astigmatism, and hyperopia with astigmatism.

### 2.5. Data Collation and Analysis

Data was collated and analysed in an Excel database. Subjects were grouped according to age, gender, presenting symptoms, and diagnosis based on the five categories identified. Descriptive statistics for categorical data included frequencies (percentages). Inferential statistics were used to make comparisons between error type, frequency of occurrence, and gender difference.

### 2.6. Statistical Analysis

The primary goal of these analyses was to determine the types and presentation of refractive errors and their possible association with gender and age as a basis for future predictions in Yemeni children and young adults especially in general practice in primary care. All analyses were performed in Microsoft Excel and an alpha level of 0.05 was considered statistically significant. Descriptive statistics of quantitative data are presented as proportions (percentages). Categorical data, for example, age group, gender, and presenting symptoms are also presented as proportions (percentages).

## 3. Results

Out of 1500 patients, 700 were males (46.7%) and 800 females (53.3%). Hyperopia was the most common single diagnosis (53.3%) followed by myopia (33.3%). Astigmatism was uncommon as a single diagnosis (13.4%) but commonly associated with hyperopia or myopia. Myopia was more common in males (42.9%) than females (25%). Hyperopia was more in females (62.5%) than males (42.9%) (Table 1). Age groups most affected by refractive errors were 13–18 years (27.7%), 19–24 years (24.8%), and 25–30 years (24.6%), respectively. Decreased vision (53%) was a common presentation in myopia and astigmatism (41.5%) and less in hyperopia (39.6%). Headache was common in astigmatism (56%), hyperopia (28.8%), and myopia (17.8%). Muscle imbalance, namely, exotropia (27.2%) is mainly found in myopia and esotropia (24.3%) in hyperopia (Tables 1 and 2).

Myopia was diagnosed in 500 patients and occurred in all age groups from 0 to 30 years but was more common in males (42.9%), compared to females (25%). There was also a progressive increase in the frequency of myopia with age from 0–6 years (28%) to 25–30 years (47.3%) (Table 1).

In contrast, hyperopia was more common among patients, 800 cases with a female preponderance (*M* = 42.9%; *F* = 62.5%). In terms of age distribution, all age groups 0–30 years were affected but highest in the 13–18-year-old group (67.8%) followed by the 19–24-year-old group (45.7%) (Table 1).

Astigmatism was diagnosed in 200 patients with increased distribution in males (14.3%) and females (12.5%). No cases of astigmatism were found in the 0–6-year-old group. It was much higher in the 19–24-year-old group (18%) compared to the other groups (Table 1).

In terms of presentation, decreased vision (53%) was a major complaint in patients with myopia with a progressive increase in frequency from age 0–6 years from 48.6% to 57.2% in the 25–30-year group. A similar trend was found in hyperopia but starting from a higher frequency of 33.3% in 0–6 years with a plateau of around (47.9%) in the age group of 25–30 years. In astigmatism, decreased vision was not found in the 0–6-year group and was less common before the age of 13 years but was a major complaint in the 25–30-year group (45.5%) and followed by 19–24-year groups (44.8%) (Table 2).

Headache was reported less in the 7–12-year-old group (9.2%) with myopia, increasing in the 25–30-year-old group (23.4% highest). In hyperopia, headache was most reported in patients between 13 and 30 years amongst whom the 19–24-year group (44.1%) complained the most followed by the 25–30-year group (40.7%) and 13–18-year-old group (34%). In astigmatism, there was a progressive decrease and increase in headache from 7 to 30 years old: 7–12 (57.1%), 13–18 (58.1%), 19–24 (55.2%), and 25–30 (54.5%), respectively (Table 2).

In our population, muscle imbalance, namely, exotropia, a form of strabismus where the eyes are deviated outward was found in 27.2% of myopia patients, and esotropia, a form of strabismus in which one or both eyes turn inward, was found in 2% of myopia patients. In myopia patients, exotropia found in all age groups 0–6 (34.3%) and 7–12 (46.1%) and 13–18 (38.9%), 19–24 (18.5%) and 25–30 (19.4%) respectively. A similar trend but much lesser frequency of exotropia was found in patients with hyperopia with the highest frequency (9.3%) in the 25–30-year group. Exotropia was not a feature of astigmatism, except in a very small percentage of the 7–12-year-old group (14.3%) (Table 2).

Similarly, esotropia was not found in astigmatism and only found in a small proportion of patients with myopia (2%). None of those aged between 13 and 30 years with myopia presented with esotropia. However, esotropia was a common feature in hyperopia, most common in the much earlier age groups 0–6 (61.1%) and 7–12-year-old group (51.7%). It was also found in the 13–18-year-old (21.4%) and 19–24-year-old group (8.8%) but very uncommon in the 25–30-year-old group (2.1%) (Table 2). Presbyopia was not reported in our population due to the age distribution as it affects most people over the age of 35.

## 4. Discussion

Refractive errors are the most common ocular problem affecting all age groups and considered as a public health challenge. Recent studies and WHO reports indicate that refractive errors are the first cause of visual impairment and the second cause of visual loss worldwide as 43% of visual impairments are attributed to refractive errors [20]. A review study showed that uncorrected refractive errors were responsible for visual impairment in 101.2 million people and blindness in 6.8 million people in 2010 [21]. Generally, refractive errors prevalence vary among the different population due to differences in their genetic background and diverse environmental factors [22]. This study determined the types and presentation of different refractive errors among children and young adults of age 0–30 years who visited the major referral center for eye problems in Sanaa, Yemen. Myopia, hyperopia, and astigmatism were common conditions affecting the ophthalmic health of the Yemeni population. We found hyperopia was the most common single diagnosis (53.3%) followed by myopia (33.3%) among this population and astigmatism was uncommon as a single diagnosis (13.4%) but commonly associated with hyperopia or myopia. Myopia was more common in males (42.9%) and less in females (25%); however, hyperopia was more in females (62.5%) and less in males (42.9%). Age groups most affected by refractive errors were 13–18 years (27.7%), 19–24 years (24.8%), and 25–30 years (24.6%), respectively.

There were not any previous publications regarding the types and presentations of refractive errors in Yemen and neighboring countries. Previous studies reported a decrease in myopia and an increase in hyperopia with increasing age [3, 23–26]. In this study, there is an increase in the frequency of myopia with age from 0–6 years to 25–30 years and was more common in males compared to females. In a study conducted on the Mexican population, myopia was the most common refractive error, and the proportion seemed to increase among the younger population (10 to 29 years old), but hyperopia increased among the aging population (40 to 79 years old) [24]. In our study population, hyperopia was the most common single diagnosis (53.3%) among age groups from 0 to 30 years and is present most with the age group of 13–18 years. This difference may be due to the age distribution of our study population. Our sample size only represents children and young adults. This needs to be considered further.

According to a meta-analysis conducted in the Middle East region, the prevalence of astigmatism was 15% (95% CI 10, 19) in subjects less than or equal to 15 years and 24% (95% CI 16, 31) in those older than 15 years of age [27]. The prevalence of astigmatism in males and females less than or equal to 15 was 9.0% (95% CI 0.7–17.3) and 9.9% (95% CI 1.5–18.3), respectively, and the prevalence in males and females over 15 years of age was 31.1% (95% CI 18.7, 43.6) and 29.6% (95% CI 17.2, 42.1), respectively [27]. We found astigmatism was uncommon as a single diagnosis (13.4%) but commonly associated with hyperopia or myopia. Astigmatism is higher in males (14.2%) compared to females (12.5%) and is associated with hyperopia and myopia. This needs to be explored further.

A review study found a higher prevalence of hyperopia in Eastern Mediterranean children than in African, Southeast Asian, and Western Pacific children [24]. A general overview of the results of a meta-analysis done by Khoshhal et al. in the Middle East reports that the Middle Eastern countries are more prevalent to have hyperopia than other parts of the world [27]. Our study also reports the same. It is likely that the Middle East may be far behind the rest of the world, especially the East Asian countries, in terms of the epidemic of myopia. The most important hypothesis seems to be that, in addition to the racial differences of this region of the world, it should be pointed out that the economic conditions of this region in the world may have caused the region not to be myopic as elsewhere in the world. Regarding a previous report, those who live more on the basis of near work or indoor activity are more prone to myopia [27].

The association of gender with refractive errors has not been well established. Some studies have reported that the prevalence of myopia is higher in men than in women [19, 28–31]. In other studies, however, this trend was not observed [32, 33]. In this study, the distribution of myopia is higher in men (42.9%) than in women (25%). Symptoms like decreased vision and headache distributed as decreased vision were relatively and more uniformly high in myopia. However, in hyperopia, decreased vision, there was a progressive decrease with age, the highest being in young adults, 19–30 years old. In astigmatism, decreased vision, there was a progressive increase in presentation with age, the highest being in young adults, 19–24 years old. Headache was more common above 12 years in myopia, hyperopia, and astigmatism conditions. Fewer patients with myopia complained of headaches, with a peak age group of 25–30 years. Patients of 13 years and above with hyperopia presented with headaches and those who are 13–18 years old complained most. Children 7–12 years old with astigmatism complained most of headaches. And conditions like exotropia and esotropia were fewer common complaints in all three conditions and at different ages. However, patients with myopia were more likely to also have exotropia, especially older children 7–12 years. In hyperopia, exotropia is high in the 13–18 year-old group and those with astigmatism rarely presented with exotropia, except for children 0–12 years old. On the contrary, esotropia was most common in children 0–12 years old with hyperopia and rare among those with astigmatism and myopia beyond 6 years and 12 years, respectively.

The limitations of the current study included that the selection of individuals was conducted using nonprobability sampling and did not also consider regional and occupational differences. For wider use and predictive generalization, a more randomized probability population sample may be necessary. Thus, despite possible limitations, a sample of patients who visited the ophthalmic clinic for any laboratory service or visual examination from different parts of the Yemen was used for the examination of refractive errors, which ensures some representation of the population. Furthermore, the types and presentations of refractive error in children and young adults have not been previously studied in Yemen. To our knowledge, this is the first report of refractive error in the Yemeni population.

## 5. Conclusion

The information of this study can be used to characterize and potentially predict types of refractive error among Yemeni children and young adults. The findings may be found useful among primary care and general practitioners and eye specialists and could help in the development of simple diagnostic tools such as clinical algorithms to aid early diagnosis and management.

## Figures and Tables

**Table 1:** Age and sex distribution of different types of refractive errors in a Yemeni population 0–30 years (*N* = 1,500)

Subgroup	Sample	Myopia (*N* = 500)		Hyperopia (*N* = 800)		Astigmatism (*N* = 200)	
		*N*	%	*N*	%	*N*	%
*Age group*							
0–6	125	35	28.0	90	72.0	0	0
7–12	220	65	29.5	120	54.6	35	15.9
13–18	413	90	21.8	280	67.8	43	10.4
19–24	372	135	36.3	170	45.7	67	18.0
25–30	370	175	47.3	140	37.8	55	14.9

*Sex*							
Male	700	300	42.9	300	42.9	100	14.3
Female	800	200	25.0	500	62.5	100	12.5

Total	**1500**	**500**	**33.3**	**800**	**53.3**	**200**	**13.4**

**Table 2:** Frequency of presenting symptoms and signs (%) for all types of refractive error according to age group (*N* = 1,500).

	Age group	Sample	^*∗*^DV		^*∗*^HA		^*∗*^Ex		^*∗*^Es	
			*N*	%	*N*	%	*N*	%	*N*	%
Myopia	0–6	35	17	48.6	0	0	12	34.3	6	17.1
	7–12	65	25	38.5	6	9.2	30	46.1	4	6.2
	13–18	90	45	50	10	11.1	35	38.9	0	0
	19–24	135	78	57.8	32	23.7	25	18.5	0	0
	25–30	175	100	57.2	41	23.4	34	19.4	0	0
	Total	500	265	53	89	17.8	136	27.2	10	2

Hyperopia	0–6	90	30	33.3	0	0	5	5.6	55	61.1
	7–12	120	50	41.7	3	2.5	5	4.1	62	51.7
	13–18	280	100	35.7	95	34	25	8.9	60	21.4
	19–24	170	70	41.2	75	44.1	10	5.9	15	8.8
	25–30	140	67	47.9	57	40.7	13	9.3	3	2.1
	Total	800	317	39.6	230	28.8	58	7.3	195	24.3

Astigmatism	0–6	0	0	0	0	0	0	0	0	0
	7–12	35	10	28.6	20	57.1	5	14.3	0	0
	13–18	43	18	41.9	25	58.1	0	0	0	0
	19–24	67	30	44.8	37	55.2	0	0	0	0
	25–30	55	25	45.5	30	54.5	0	0	0	0
	Total	200	83	41.5	112	56	5	2.5	0	0

^*∗*^DV = decreased vision; HA = headache; Ex = exotropia; Es = esotropia.

## Data Availability

The datasets generated and/or analysed during the current study are not publicly available but are available from the corresponding author on reasonable request.
